# Extra-institutional science: DIY biologists’ democratization of scientific practices and spaces

**DOI:** 10.1057/s41292-024-00347-w

**Published:** 2025-01-28

**Authors:** Anna Verena Eireiner

**Affiliations:** Department of Sociology, University of Cambridge, Free School Lane, Cambridge, CB2 3RF UK

**Keywords:** Do-it-yourself biology, DIY biology, Community laboratories, Extra-institutional science, Alternative epistemic spaces, Alternative scientific practice

## Abstract

DIY biology, or Do-It-Yourself biology, refers to a movement where individuals and communities establish laboratories outside traditional academic and industrial settings—such as in garages, kitchens, or community spaces. DIY biologists experiment with gene-editing technologies like CRISPR, cultivate glow-in-the-dark plants, and engineering colorful fungi. This practice challenges established norms in research, advocating for decentralized and community-driven approaches to scientific inquiry and innovation. DIY biologists are often trained scientists who choose to conduct their research in community or home laboratories. The DIY biology movement highlights that science’s boundaries are flexible and sometimes ambiguous (Gieryn in Am Sociol Rev 48:781–795, 1983). By operating outside traditional research institutions, DIY biologists challenge established authority, hierarchies, funding structures, and proprietary regimes. They create a distinct identity beyond the increasingly neoliberalized institutional spheres of modern knowledge production, showcasing alternative ways to pursue science. I theorize DIY biology as ‘extra-institutional science’ due to its emergence outside conventional laboratories of industry and academia. This research draws on empirical data from interviews with DIY biologists and the 2021 DIY Biology Community Survey.

## Introduction

The DIY biology movement emerged in the early 2000s, in US cities, such as Boston, San Francisco, and New York (Landrain et al. 2013; Meyer 2014). DIY biology laboratories then took roots in other cities and countries, making a home for community scientists aiming to democratize biotechnology and explore DIY experimentation (Roth 2019; Tocchetti 2014). DIY biology is known by various terms (e.g., biohacking, community biology, garage biology). In this paper, the movement is referred to as ‘DIY biology,’ as the majority of research participants used this term and self-identified as DIY biologists. The term was consistently used when recruiting participants for interviews and the survey.

DIY biology community members retain core scientific practices but operate outside of corporate and academic research institutions, thereby crafting alternative epistemic spaces. Similar to policymakers concerned with assessing the movement, DIY biologists engage in so-called ‘boundary work’ (Gieryn 1983) around the question of DIY biology’s scientific potential vis-á-vis traditional research institutions. The attempt to define the boundaries of science is one of the oldest themes in the philosophy of science. The DIY biology movement is a reminder that science is not a natural or essential entity; “its boundaries are drawn and redrawn in flexible, historically changing and sometimes ambiguous ways” (Gieryn 1983: 781). This research investigates how DIY biologists (re-)draw these boundaries by envisioning and realizing alternative epistemic spaces. For this purpose, I draw on data from 23 personal interviews with DIY biologists and the 2021 DIY Biology Community Survey (*n* = 152) that I designed for this project. Furthermore, I situate, contrast, and contextualize my findings within existing scholarly accounts of DIY biology.

In the first part of this article, I shed light on the institutions of scientific knowledge production to which DIY biology has an ‘extra’ relationship. Specifically, I argue that DIY biology, as an extra-institutional science movement, does not operate entirely independently from traditional research institutions. Instead, the movement’s relationship with academia and industry is characterized by various entanglements, e.g., in employment, training, spaces, and supply chains. This paper focuses specifically on extra-institutional science vis-á-vis academia and industry. I consider these institutions characterized as formal organizations and locations of scientific knowledge production (Endruweit et al. 2014: 366–368). Institutions can also be conceptualized differently, e.g., as systems of norms and values (ibid.). These conceptualizations are valuable but beyond the scope of this analysis. Here, I particularly focus on the empirically under-investigated relationship between DIY biology and the academy and argue that DIY biology can be thought of as the product of academic research institutions in crisis.

The second part introduces three socio-technical imaginaries (Jasanoff and Kim 2015) that emerged from the data. First, DIY biologists see their movement as a way to explore socially relevant research agendas. Second, they envision democratizing the scientific enterprise. Third, they argue for greater intellectual freedom in extra-institutional science compared to traditional academia. These imaginaries shed light on the fundamental tensions in DIY biology’s boundary-challenging activities.

The final part focuses on DIY biology’s economies, explaining how DIY biologists create extra-institutional spaces and communities outside traditional funding regimes and supply chains. The research argues that DIY biology’s principles clash with the need to fund and supply these spaces under the current economic system. It introduces three initiatives as examples of DIY biology success, illustrating how the movement is intertwined with and may even accelerate capitalist economies.

## Research methods

The following insights build on scholarly accounts, my 2021 DIY Biology Community Survey data and findings from 23 personal interviews with DIY biologists.

I conducted 23 semi-structured interviews with DIY biologists between May 2020 and September 2022. I selected relevant interviewees by (virtually) attending networking events on the topic of DIY biology (e.g., the Global Community Biosummit 2020, 2021). I also recruited interview participants in person at DIY biology community laboratories and events in the UK, Germany, and Canada. Additionally, some participants were referred to me by other interviewees. This process introduces a selection bias, as it likely favored more extroverted, well-networked individuals active in international contexts and in the countries I visited. DIY biologists with home laboratories or more individual practices may have been underrepresented.

Potential interview partners received a brief introduction to the project. If they decided to take part, they were asked to sign an informed consent form.

The participants in my study ranged in age from their early twenties to late fifties, with many concentrated in their late twenties and early thirties. There was an almost equal gender split, and the majority were highly educated, holding advanced degrees in the natural sciences. The age distribution and educational backgrounds closely resemble existing demographic data on DIY biology community members (e.g., Grushkin et al. 2013).

I chose semi-structured interviews to ask the same open-ended questions while having the flexibility to branch out and explore topics further (e.g., Crewell 2014: 319; Baur and Blasisus 2014). The interview questions were not always presented in the exact same order, and participants were encouraged to elaborate as they saw fit (Baur and Blasisus 2014: 53). Conducting research during COVID-19 affected the data collection process, sometimes limiting in-person interactions. Additionally, it may have influenced the dynamics of interviews conducted virtually, potentially leading participants to be less open than they might have been in face-to-face settings.

Interviews were either conducted remotely via video chat or in-person. I audio-recorded interviews with interviewees’ prior consent. I asked DIY biologists about their motivations and challenges. I also inquired about their backgrounds in academia and industry as well as potential connections with regulators and sponsors.

I transcribed interview recordings with the assistance of the transcription software Descript. The resulting transcripts were then coded using the software tool NVivo. I added codes for DIY biologists’ relationship with traditional research institutions, entrepreneurial aspirations, challenges, and experiences with regulators and other stakeholders. I consolidated these codes in various iterative rounds.

I anonymized participants’ and interviewees’ personal information by omitting personal identifiers and utilizing generic descriptors instead (i.e., Community Member, Interviewee #8). I informed interviewees of this anonymization procedure while establishing informed consent.

The 2021 DIY Biology Community Survey, a pivotal part of this research, was active from March to November 2021, featuring 28 questions encompassing various response formats. Conducted using Qualtrics survey software, it was distributed through platforms like Twitter, Facebook, Slack, as well as DIY biology-specific email lists and meet-up groups. Of the 154 respondents, 152 completed multiple items, contributing to the final dataset. While not fully representative of the global DIY biology community, with over 5100 members in the DIYbio Google Group (2022), the survey provides valuable insights into the community’s landscape. It also explores less-discussed themes, such as DIY biology’s connections with academia and industry. Participation was open to all self-identified DIY biologists, ensuring diverse perspectives were captured.

DIY biologists were presented with an informed consent statement, which included information on the purpose of the study, duration, and risks. Survey data were filtered, classified, merged, and cleaned using Qualtrics. The codes developed through the interview data was also applied to survey data.

This article draws primarily from a survey section on DIY biologists’ relationships with academia and the life sciences industry. Respondents were asked about their current or past employment in these fields. Those who indicated academic employment were asked additional questions about their job title and whether they had considered leaving academia for full-time DIY biology. Depending on their response, participants wrote a short essay on their preference for academia or DIY biology.

Only respondents who reported never being employed in academia (*n* = 68) were asked about their employment in the life sciences industry or a related field. This decision aimed to maintain survey brevity and engage respondents effectively. Additionally, focusing on the academia‒DIY biology relationship, an area that has received less empirical attention, was a deliberate choice.

In interviews, I posed initial questions and followed up to clarify or allow interviewees to elaborate on their experiences in academia and/or industry. This data informs the discussion on DIY biology’s intricate connections with academia and industry, as well as the complex economies of extra-institutional science.

## DIY biology as extra-institutional science

Commentators in academia and policy circles often characterise DIY biology as ‘citizen science’ (e.g., Sauter et al. 2015). However, several authors have pointed out that the DIY Biology movement does not neatly fit within existing definitions of citizen science. Citizen science is commonly understood to encompass participatory practices aimed at including non-professionals (or ‘amateurs’ or ‘laypeople’) in the enterprise of scientific knowledge production (Bonney et al. 2014; Strasser et al. 2019).^1^ While DIY biology and citizen science share some similarities, they are not entirely aligned. Both DIY biology and citizen science exhibit a techno-optimist outlook at times. They both provide unique opportunities for individuals to actively participate in, and contribute to, scientific projects. However, citizen science typically does not venture into the realm of biosafety and biosecurity. Citizen science is often perceived as a pedagogic endeavor and, unlike DIY biology, does not evoke concerns about potential biosafety hazards. Moreover, citizen science and DIY biology projects typically carry different objectives, thus entailing different implications and stakes.

While Bonney et al. (2014) and Strasser et al. (2019) conceptualize citizen science as participatory practices that include laypeople in scientific knowledge production, DIY biology represents a more professionalized form of participation. My concept of ‘extra-institutional science’ builds on and diverges from these frameworks by emphasizing the professional expertise and organizational structures of DIY biology, which operate outside traditional institutions.

In this context, ‘professionalized’ implies that individuals in extra-institutional science often possess formal qualifications or expertise and their practices reflect a structured, specialized approach as it is found in traditional research institutions. DIY laboratories are typically set up and populated by individuals with degrees in the natural sciences, i.e., the same group of people one finds in academic, industrial, and government laboratories and would thus be considered as biotechnology experts. A quantitative study conducted by Grushkin et al. (2013) supports this argument: 61.9% of DIY biologists were instructed in biology at a college or university level, with 15.1% having studied it at the doctoral level. The results of my 2021 DIY Biology Community Survey support these findings. 92% of the respondents claimed to have at least some college-level education, with 76% either holding or currently studying toward a degree in the natural sciences (DIY Biology Community Survey, 2021).

Some DIY biology projects have a commercial orientation, which constitutes another difference with the conceptualization of DIY biology as citizen science, the latter of which does not assume that citizen scientists generally transform their epistemic outputs into commercialized and/or for-profit technologies (e.g., Delfanti 2013; Gorman 2011).

Eglash’s concept of ‘appropriated technology’ (2004a) provides a useful framework for understanding DIY biology as an example of technological adaptation by communities operating outside traditional institutions. While Eglash (2004b) focuses on technoscientific (re-)invention within and by underrepresented communities, DIY biology appropriates biotechnological tools and practices, illustrating how extra-institutional science can generate novel forms of scientific knowledge beyond established hierarchies. Like Eglash, this work examines research conducted outside the professional realms of high-tech laboratories.

Here, ‘institutional’ science refers to research carried out within traditional settings such as academia, industry, and government labs, where formal structures, professional hierarchies, and established protocols shape the production of scientific knowledge. In contrast, extra-institutional science is imagined, practiced, and (re)invented within what Eglash describes as “a space of flows”—a concept that extends “beyond geographic locations to encompass the networks of information and material exchanges that increasingly define a knowledge-based economy” (Eglash 2004b: 2). Specific physical locations, such as community or home laboratories, are key to DIY biology. These spaces are always enmeshed in a larger flows of collaboration, resources, and knowledge. Unlike traditional scientific laboratories, these flows are not institutionally formalized (e.g., supply chains) but operate in decentralized and informal ways. Extra-institutional science flows often follow open-source principles and are composed of online communities and peer support that offer knowledge sharing that transcends geographic boundaries, a global sourcing of supplies, and cross-country, virtual collaborations.

Another way in which DIY Biology has been conceptualized is as a hacking or making community (e.g., Keulartz and van den Belt 2016: 3; Levy 2001). Indeed, the DIY biology movement’s entanglements with the hacker movement are technical and spatial, as the tools and physical spaces of hackerspaces (Davies 2017) and DIY labs are often shared and semantic (‘biohacker’). DIY biologists also loosely share a ‘hacker ethos,’ which foregrounds “sharing, openness, decentralization, free access to computers or tech, and world improvement” (Keulartz and van den Belt 2016: 3; Levy 2001). DIY biologists similarly share a ‘making-do’ ethos and redesign equipment in simpler, more affordable but functional forms (i.e., Open Wet Ware, n.d.; SenseLab 2018).

According to Davies (2017), DIY biology can be thought of as less diverse and more centralized than the hackerspace movement. I argue that the exclusivity of the DIY biology communities and their degree of professionalization distinguishes them from most maker and hacker communities and citizen science endeavours. The ‘DIY’ in DIY biology is more indicative of the community's ideal to ‘make-do’ with few resources than of an amateurization of biotechnology. Even though DIY biologists seek independence from academic science, they remain closely entangled with it. As noted above, many DIY biologists hold academic degrees and/or have ties to an academic research institution. Furthermore, 21% of my 2021 DIY Biology Community Survey participants report that they are currently employed in academia, while another 35% have been employed in academia in the past.

Toupin’s (2014) feminist critique of hacking, which addresses issues of gender and inclusivity in hacker communities, contrasts with the professionalized and often somewhat exclusive nature of DIY biology communities. While hacker and DIY biology movements share some commonalities, such as decentralization and open access, the latter’s focus on professional expertise and biotechnological innovation differentiates it as a form of ‘extra-institutional science,’ a concept that moves beyond Toupin’s anarchist framing of hacking.

Kelty’s (2010) notion of ‘outlaw biology’ encapsulates how DIY biology communities operate beyond institutional boundaries. By engaging in biotechnology outside formal regulatory structures, these communities create alternative spaces of scientific inquiry, which aligns with my conceptualization of ‘extra-institutional science’ as a space that challenges traditional oversight mechanisms. Extra-institutional science focuses on professionalized scientific practices organizing and operating outside traditional institutions. Outlaw biology has a broader scope that involves challenging regulatory norms and established scientific practices often with less formal organization.

Thus, this research holds that DIY biology should be understood as an alternative research avenue outside of traditional research institutions. My concept of ‘extra-institutional science’ specifically addresses this by capturing professionalized communities and initiatives that create scientific knowledge outside of the established triad of academic, industry, or government research institutions. Unlike citizen science or amateur-led research, extra-institutional science is rooted in professional expertise but operates in alternative epistemic spaces, challenging conventional scientific boundaries while maintaining core scientific practices. In doing so, extra-institutional science movements craft alternative epistemic spaces that challenge the boundary between science and the publics as well as the hierarchies, authorities, funding regimes, oversight structures, practices, and proprietary regimes of traditional spaces of scientific research.

Extra-institutional science does not radically modify or challenge scientific methods but offers and alternative organizing structure for scientific exploration. One could argue that that this presents an inherent tension: By defining itself in relation to existing institutions, extra-institutional science is on track to become another sort of organizational structure. For instance, one could argue that community laboratories are in the process of becoming stable and formalized organizations in their neighbourhoods and cities, serving educational, and scientific purposes. As such, they are in the process of establishing rules, norms, and procedures (e.g., for biosecurity and -safety) that guide their operations. In the process of becoming institutionalized, they are recognized and supported by and within their formal legal and political systems. As such they may form a fourth institutional locus of scientific inquiry besides academia, industry and government laboratories.

While extra-institutional science captures this process of evolving organizational stability, it describes initiatives in the sphere of DIY biology and is also well suited to conceptualize many more professionalized initiatives under the umbrella of hacking or making, e.g., the MakerBot project (Makerbot, n.d.). The term advances both theoretical understanding of and practical considerations regarding the implementation of DIY biology. Understanding DIY biology as extra-institutional science furthermore acknowledges the movement’s impact and allows us to pay attention to DIY biology communities’ expectations, values, and goals in the shaping of biotechnology in general and in researching the novel scientific field of synthetic biology more specifically.

In the literature, DIY biology has been described with various terms, including ‘garage biology’ (Carlson 2005), ‘backyard biology’ (Tocchetti 2014), ‘biohacking’ (Delfanti 2013; Wohlsen 2011), ‘outlaw biology’ (Kelty 2010), ‘amateur biology’ (Delgado 2013), ‘biopunk’ (Patterson 2010; Wohlsen 2011), and ‘kitchen biology’ (Wolinsky 2009; Jen 2015). Some of these terms are used interchangeably with ‘DIY biology’; ‘biohacking’ is one example. ‘Garage biology,’ ‘backyard biology,’ and ‘kitchen biology’ all emphasize the extra-institutional nature of DIY biology activities. Other terms, such as ‘outlaw biologist’ or ‘biopunk,’ highlight the movement’s unconventional or rebellious tendencies.

Other terms offer novel conceptualizations of the movement. I want to highlight one of these concepts: ‘fringe biology.’ Vaage (2017) coined the term ‘fringe biology’ to describe “the heterogeneous, multifaceted array of societal and cultural approaches to biotechnology” (Vaage 2017: 11). The term is introduced to capture actors that use biotechnological methods for non-scientific purposes, such as bioart, which have sometimes been overlooked (Vaage 2017). ‘Fringe biology’ thus offers a useful addition to the terminology.

While fringe biology and extra-institutional science may go hand-in-hand at the same DIY laboratory, Vaage’s term focuses on those individuals who may not have any prior training in the natural sciences. *Fringe biology* specifically refers to art and design practices and citizen science endeavours that fall under the umbrella of DIY biology. In contrast, *extra-institutional science* defines professionalized scientific inquiry outside of established institutions.

However, it should be noted that many DIY biologists aim to move beyond the rigid binaries, such as science‒nonscience, expert‒amateur, and science‒publics. For instance, DIY biology community laboratories are open to publics. Many community laboratories host artistic and educational workshops in the same space that is home to aspiring biotech start-ups (e.g., LaPaillaise 2022). Moreover, DIY biologists aim to make biotechnology and its tools accessible to the average citizen (Patterson 2010). The aim of my conceptualization of DIY biology as ‘extra-institutional science’ is not to reinforce any binaries but to draw attention to an expert community that has, unbeknownst to many, moved beyond the institutional bounds of industry and academia to avoid the restrictions placed on science within traditional institutions.

## Moving beyond traditional research institutions

Existing scholarly accounts offer some helpful perspectives on DIY biology’s relationship with the modern academy and industry. Results from the 2021 DIY Biology Community Survey I conducted indicate proximity between DIY biology and traditional research institutions that previous scholarly accounts observed but did not put into numbers (e.g., Delfanti 2012; Wexler 2015). This article holds that DIY biology, as an extra-institutional science movement, does not operate completely independently from traditional research institutions. Instead, the movement’s relationship with academia and industry is characterized by various entanglements, e.g., through employment, training, spaces, and supply chains. I argue that in many ways, DIY biology is indicative of a crisis in academia sparked by increasing neoliberalization. As traditional research institutions become high-pressure environments, DIY biology emerges as a response to these shortcomings, offering an alternative epistemic space outside the academy.

### DIY biology as an alternative epistemic space

In this context, extra-institutional science can be understood as a response to the crisis facing academic research institutions. Simons (2021) asserts that DIY biologists “wish to preserve the insights and methods of science, but free it from what they see as the straightjacket of the institutionalized role of the academic scientist” (p. 156). Other studies on this topic show that scientists often turn to DIY biology as an alternative to academia, seeking to escape its hierarchies, rigid research agendas, competitiveness, and precarious employment (e.g., Delgado and Callén, 2017; Ferretti 2019). Delfanti (2013) emphasizes that DIY biology represents a form of open science, resisting the privatization and commercialization of academic research while promoting a more democratized and accessible approach to scientific knowledge. Similarly, Wohlsen (2011) underscores how DIY biologists utilize unconventional spaces, such as garages and community labs, to pursue research beyond the confines of universities and corporations.

**Fig. 1:**
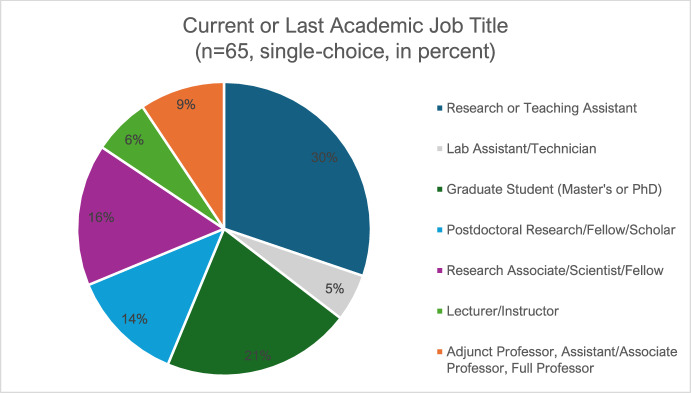
DIY Biology and Careers in Academia. This question was answered by a subset of respondents who indicated prior employment in academia, Data: 2021 DIY Biology Community Survey

Delfanti and Söderberg (2015: 13) assert that a “substantial segment of the global do-it-yourself community” holds jobs “in an academic or government lab” (Delfanti and Söderberg 2015: 13). As explained previously, my 2021 DIY Biology Community Survey data support this assertion (see Fig. 1): 57% of the survey participants indicate past or present employment in academia. Additionally, the survey indicates that participants are most often employed as research or teaching assistants (29%), graduate students (20%), research associates (15%), and postdoctoral researchers (12%).

Participants in the 2021 DIY Biology Community Survey who indicate that they were employed in academia (present or past) were asked if they prefer to work in DIY biology or academia full time: 34% of participants either left or consider leaving academia to work in DIY biology full time. Another 46% had either left or consider leaving academia for other reasons. Numerous respondents who replied that they would rather stay in academia (20%) state that they are doing so for financial reasons, as they would not be able to support themselves as full-time DIY biologists.

Many of the respondents give detailed, passionate responses when asked how they think DIY biology compares to academia. There is evidence that DIY biology community members consider DIY biology as a valid alternative to academic research. Some survey participants consider DIY biology a more promising avenue for scientific problem-solving:

Knowledge is a set of tools used on problems. Academia shows the science of the tools and a little of its application or philosophy. DIY biology is about solving questions and acquiring tools for that purpose. […] The innovations that will solve major world problems will not happen in a vacuum […], but as a labour of love in a garage/home lab.

The above statement is a good example of how DIY biologists perceive their movement as a departure from traditional institutional research. This DIY biologist implies that academic research is too narrow to solve major world problems. DIY biology is imagined as the application of academic knowledge and tools to solve urgent problems.

When asked why they did, or potentially would, leave academia to pursue DIY biology full time, one 2021 DIY biology community survey participant produced a particularly emotional response:

Corruption, mobbing, bullying, financially motivated publications, theft of ideas, totalitarianism from the leadership […] no openness to candidates, violation in the metadology [sic] of recording experiments, falsification of results, and much more that turned the world’s science into a stinking farce.

This statement is just one example of the many passionate responses produced by survey participants. An overall analysis of the open-ended responses 2021 DIY Biology Community Survey participants produced reveals several commonly shared push factors: Participants most often mention repetitive tasks and limited freedom to pursue their own research interests as an unfavorable factors in academia. The next most important factor was academic leaders who are perceived as corrupt/abusive of their powers and primarily financially motivated when setting research agendas. Other factors, in descending order, are steep hierarchies, an unhealthy, hypercompetitive workplace atmosphere as well as a pressure to produce publications (‘publish or perish’).

These statements bear witness to the process of neoliberalization that is radically altering the experiences of staff and students in contemporary higher education. The neoliberalization of the academy implies a reorganization according to economic principles. The process of neoliberalization prioritizes bottom lines and reorders faculties and departments as business units (Fraser and Taylor 2016). This is to say that profitability becomes a decisive factor in the reorganization of the academy. Since the 1990s, institutions in higher education are increasingly seeking to improve their branding to compete in international rankings (Fraser and Taylor 2016). A part of this quest for competitiveness is high publication output, which leads to the ‘publish and perish’ aphorism that DIY biologists criticize and seek to escape from. While tuition costs are deregulated and increased, academic labor is undergoing intensification and casualization (Collini 2012; Fraser and Taylor 2016; Heath and Burdon 2013). Many academics face precarious contracts, low wages, fierce competition, immense pressures, and high student-to-staff ratios (Fraser and Taylor 2016; Health and Burdon 2013). This arguably leads to the unhealthy workplace atmosphere that DIY biologists identify as grievances/push factors.

### Entanglements: DIY biology and academia

Despite these grievances, it is somewhat unexpected that DIY biology remains closely intertwined with academic institutions in various ways. One example is spatial proximity to academic research institutions. My 2021 DIY Biology Community Survey shows that DIY biologists most often work at a community lab (53%) and their private home lab (50%) (See Table 1). However, the data also indicate that some DIY biologists practice within institutional bounds. This is to say that 20% of participants state that they work on their projects at an academic lab at a university or school, while 13% use a company lab. About half of the participants who work at academic labs utilize other locations as well, namely community labs, home labs, and university-based DIY biology labs. While more and more DIY biology labs are setup at the fringes of universities, only 5% of DIY biologists indicate that they work at a university-affiliated DIY biology lab.

**Table 1:** DIY biology spaces and places

Where do you carry out your DIY Biology work?	Multiple choice (*n* = 107) %
Independent community lab space	53
My private home lab space	50
Academic lab, such as university or school lab	20
Company lab	13
Shared home lab space (e.g. garage)	10
University-based DIY biology lab	5

Data: 2021 DIY Biology Community Survey

Although practicing DIY biology at an academic institution seems paradoxical, DIY biologists seem to manage this tension well. In a personal interview, one DIY biologist explains to me that they find it practical to use slow days or after-hours at their academic research job to complete lab work for their DIY biology project (Community Member, Interviewee #19). Their motivation for working in DIY biology is that the topic they feel particularly passionate about is disregarded within their lab’s research agenda. The interviewee deems their self-chosen project socially relevant, thus, with the blessing of their academic superior they decided to pursue the project in their spare time. The interviewee explains that they utilize the academic space and materials, while simultaneously drawing on the advice of other DIY biologists and DIY biology online resources.

Moreover, DIY biology work within institutional bounds is increasingly formalized in the shape of university-affiliated DIY biology labs. Two leading members of a university-affiliated DIY biology lab explains that setting up and running their space is much easier within the umbrella of an academic institution. It allows them to utilize protocols and infrastructures for essentials, such as waste disposal, supplies, and safety oversight (Community Member, Interviewee #10 and #11). A DIY biologist running a university-based DIY biology laboratory gave the same reasoning. They also mention that compared to an independent community laboratory, the university laboratory gives their projects more visibility and thus better access to potential sponsors (Community Member, Interviewee #23). DIY biology within or at the fringes of an institution seemingly serves both the interests and goals of DIY biologists (practical and administrative support, funding) and the state (risk management through institutional oversight).

Conversely, in personal interviews, lab leaders at university-based community laboratories say that it is more difficult to attract individuals that have no affiliation with the university. DIY biology is often imagined as egalitarian (JOGL 2023) and accessible to all (Patterson 2010), which is why the university-based DIY biology laboratory may present a tension.

In addition to the organisational and spatial proximity between institutional and extra-institutional biology, the history of the DIY biology community is intricately entangled with that of industry and academia. Besides DIY technologies and second-hand marketplaces, a steady supply through traditional research institutions were key factors in the movement’s evolution (e.g., Delfanti 2013; 2014; Kolodziejczyk 2017). Previous studies suggest that extra-institutional science laboratories could not have emerged if low-cost equipment and spare parts that allow individuals to build cheap replicas had not become more accessible (Meyer 2013; Kuznetsov and Paulos 2010).

DIY biologists rely on online tutorials and second-hand equipment, often purchased at a fraction of the original cost (Landrain et al. 2013). These tools, previously used in traditional labs, are now accessible through online marketplaces, enabling experiments that were once expensive to be replicate (Ferretti 2019: 4). DIY biologists often manage a supply through research institutions, as many suppliers only deliver to institutional laboratories. Paradoxically, even DIY BioBrick DNA kits are exclusively shipped to academic labs (Landrain et al. 2013). Additionally, DIY biologists utilize scientific knowledge from academic journals and other scholarly sources (Wexler 2015). Online forums and DIY platforms provide a community for technical support and feedback, mirroring aspects of academic peer review and replication (Kuznetsov and Paulos 2010).

### Entanglements: DIY biology and industry

The relationship between DIY biology and biotechnology markets is intricate. While this research offers fresh insights into the varied perceptions of DIY biology across different contexts, scholarly discourse has extensively examined the movement’s complex ties with the biotechnology industry, exploring how DIY biology ideals intersect, clash with, or complement market principles (e.g., Delfanti 2013; Ikemoto 2017). These diverse interpretations serve as a foundation for the empirical aspect of this study.

Among respondents to the 2021 DIY Biology Community Survey who reported never being employed in academia (*n* = 68), questions about their involvement in the life sciences industry were posed. Of these respondents, 28% are currently employed in the industry and 15% have been in the past. Those indicating industry employment were further asked if they had considered or would consider transitioning to full-time DIY biology work, with 43% expressing willingness to do so. Reasons cited for this preference include the opportunity for self-directed work, community engagement, advancing social justice, and entrepreneurship.

Respondents who preferred to remain in the industry highlighted financial stability and the ability to fund DIY biology pursuits with their industry salaries. Some also preferred maintaining their industry roles while exploring entrepreneurial opportunities within DIY biology on the side.

It was notable that DIY biologists produced much more passionate, emotional responses when asked to compare DIY biology with academia. This, and the fact that DIY biologists’ involvement in academia is thus far comparably empirically under-investigated, motivates the following in-depth investigation on the movement’s relationship with the academy.

The previous section has shown that DIY biology and traditional research institutions are, and have always been, interlinked. It is surprising, perhaps even paradoxical, that DIY biologists envision their movement as an alternative to traditional research institutions. What follows is a detailed account of the ways in which DIY biologists imagine their movement vis-à-vis the academy.

## Imagining extra-institutional science

Even though working at traditional institutions disgruntles some DIY biologists, it does not seem to diminish their love for research. Instead of attempting a revolution from the inside, DIY biologists envision extra-institutional science communities and spaces. To learn more about the movement’s identity construction vis-à-vis traditional research institutions, participants were asked to elaborate on what attracts them to DIY biology, as compared to industry corporations and the academy. In personal interviews and my 2021 DIY Biology Community Survey, participants generally imagined their movement along the lines of three themes: socially relevant research agendas, the democratization of science, and intellectual freedoms. These large-scale visions are collectively held by DIY biologists across country contexts, which is why I identify them as socio-technical imaginaries.

The first imaginary focuses on the democratization of science, where the movement advocates for open access to scientific tools and knowledge, aiming to make research more inclusive and participatory. This contrasts with traditional science, where access is often restricted to institutional settings, reinforcing exclusivity.

The second imaginary concerns intellectual freedoms, with DIY biologists valuing the freedom to explore unconventional ideas without the constraints of institutional priorities. This differs from academic and corporate research environments, which are frequently shaped by funding sources and institutional agendas.

The third imaginary focuses on socially relevant research agendas. DIY biologists emphasize research that addresses societal challenges and aims to apply scientific knowledge to practical problems. This contrasts with traditional academic research, which may prioritize theoretical outcomes over applied ones. This imaginary highlights a shift toward problem-oriented science that directly impacts communities.

I employ the socio-technical imaginaries concept to analyze how extra-institutional science is envisioned by DIY biologists. The concept of socio-technical imaginaries refers to the shared visions or collective imaginations that societies have about the relationship between science, technology, and social organization (Jasanoff and Kim 2015). Accordingly, sociotechnical imaginaries can be defined as

collectively held, institutionally stabilized, and publicly performed visions of desirable futures, animated by shared understandings of forms of social life and social order, attainable through, and supportive of, advances in science and technology. (Jasanoff and Kim 2015: 4)

The case of extra-institutional science shows that novel developments in science and technology and concomitant uncertainties are prime examples where socio-technical imaginaries are invoked and challenged. Understanding how these imaginaries influence DIY biology’s development provides insights into why certain futures are favored (The Sociotechnical Imaginaries Project 2011).

These three socio-technical imaginaries emerged through in-depth thematic analysis of both interview and survey data following the approach laid out by Braun and Clarke (2020). These recurring, cross-cutting socio-technical imaginaries offer a useful tool to better understand DIY biology’s motivations in the construction of alternative epistemic spaces. In the following, I describe and contextualize each of these socio-technical imaginaries, describing how these imaginaries are thought to run counter to established research institutions, specifically academia. Additionally, I point out potential conflicts and pitfalls that arise in the identity construction of the movement.

### Imaginary #1: democratization of science

The first socio-technical imaginary is the democratization of biotechnology. Frow (2015) observes that the calls for ‘democratization’ by DIY biologists are threefold: a call toward accessibility of research infrastructures, a challenge to the expert-publics divide and the formation of open-source ownership regimes (Frow 2015). Many scholarly accounts highlight DIY biology’s objective to openly share and circulate methods and findings (e.g., Ferretti 2019; Delfanti 2013). Rather than pursuing commercialization, this approach is believed to foster collective benefit (e.g., Wohlsen 2011; Meyer 2013).

2021 DIY Biology Community Survey participants voice that they feel strongly about all of these aspects. Many participants specifically voice that they favour and want to advance DIY biology’s non-proprietary knowledge regime. This alternative knowledge regime is thought to support the goal of socially relevant research. Instead of patenting and/or monetizing new technologies, DIY biologists seek to disseminate their findings in hopes of collective benefit. This, again, is thought to counter the neoliberal academy. In the neoliberal academy, knowledge is not freely disseminated but patented in hopes of generating new market opportunities for capitalist endeavors.

DIY biologists also advocate for accessible research infrastructures and civic engagement and education, which is thought to counter elitist structures, academic paternalism, and knowledge privatization (e.g., Delfanti 2013). Research participants voice that they seek to make hands-on science education accessible to a wider audience. One 2021 DIY Biology Community Survey participant explains how DIY biology is an empowering experience as compared to traditional science education:

I like it [DIY biology] because I get to feel and do the very thing I studied and it’s totally different from what they teach you in school. Oftentimes there’s only so much you can learn in diagrams and lectures; you really do have to do it yourself to get a feel for the feedback it takes to do real biology. And it’s even better when you get to reconstruct things yourself and you get an appreciation for how much work was done in this world.

Unlike universities, spaces of extra-institutional science may provide an open and inviting and, thus, accessible environment. Scholars observe that “in contrast to academic laboratories, the places where DIYbio is carried out usually allow access to everyone, regardless of their academic and socio-cultural background” (Landrain et al. 2013). It is often claimed that DIY biology enables new forms of civic engagement in contemporary knowledge societies that not only offer a way to train individuals in the life sciences but that also bridge the gap between expert elites and laypeople—the governors and the governed (e.g., Kelty 2010; Pauwels and Denton 2018; Tanenbaum et al. 2013). This is a theme that many research participants also picked up on. A 2021 DIY Biology Community Survey participant voice their hopes that DIY biology will lower the entry barriers to science, thereby counteracting misinformation:

Science access is a pretty big problem, as evidenced by the enormous science misinformation going around. DIY Biology exists because of the lack of science access intersecting with those who want to learn on their own. The barrier to entry is too high for most of the population to get into, which is already a telling problem. But even if they can’t become a PhD, I think accessing the tools and being able to learn the knowledge should be something that is accessible. […] If you want to culture a society that adores science, is amazed by science, and wants to pursue more of it, then I think [DIY biology community laboratories] are very good models to look at.

It could be argued that hopes of “culturing a society that adores science” glorify science. This touches upon two problems in the identity construction of DIY biology: techno-optimism and the deficit model.

First, DIY biology seemingly carries a techno-optimism that fails to fundamentally challenge the notion that science and technology innovation and entrepreneurship are necessarily of service to society. DIY biologists dedicate themselves to issues that may not get enough academic attention; yet there seems to be a tendency to ignore the social, economic, political, and cultural underpinnings of grand-scale issues, such as the climate crisis and antibiotic resistance. These grand challenges will not be solved by technological and scientific progress alone.

Second, DIY biologists occasionally employ the deficit model (Wynne 1992; Ziman 1991), which is based on the premise that laypeople or “amateurs” should acquire a scientific understanding that aligns with that of scientists (Ziman 1991). The underlying belief is that if the public possessed scientific knowledge, they would be more receptive to novel technologies. However, the deficit model overlooks the fact that science is not rigidly defined, often yields inconclusive results, and is frequently combined with other sources of judgment (Wynne 1992).

While DIY biology advocates for open access and democratization, it is crucial to acknowledge that these ideals can sometimes oversimplify the complexities involved in making science accessible. The movement’s approach to democratization, although well intentioned, might not fully address the existing socio-economic barriers that affect equitable participation in science. Furthermore, the notion of democratization within DIY biology spaces may not entirely dismantle the institutional hierarchies and exclusivities but rather reconfigure them in new forms.

### Imaginary #2: intellectual freedom

The second socio-technical imaginary guiding DIY biology is personal freedom and self-development. The movement is characterized by an aversion toward rigid research agendas and the tediousness of only independently running a small fragment of an academic research group’s project. Compared to institutional science, the extra-institutional laboratory seemingly promises a “curiosity-driven approach” (Ferretti 2019: 4), independence, and agency. DIY biologists imagine that community laboratories will give them a setting in which they can define their goals, play, learn, experiment, observe, direct and redirect their foci (Delfanti 2011: 55).

In her ‘Biopunk Manifesto,’ community leader and visionary Meredith Patterson poignantly articulates DIY biology’s central concerns regarding institutional science and compares an individual’s freedom of inquiry to freedom of speech and religion:

We reject the popular perception that science is only done in million-dollar university, government, or corporate labs; we assert that the right of freedom of inquiry, to do research and pursue understanding under one’s own direction, is as fundamental a right as that of free speech or freedom of religion (Patterson 2010).

Meredith Patterson’s statement makes clear how much value DIY biologists place on individual freedom to research and learn. DIY biology centres the individual, allowing them to explore scientific methods and set their personal agendas. Moreover, participants voice that they value DIY biology’s approach to independent and community-led learning and its open-ended, curiosity-driven approach. These principles are imagined to reorder research in a way that favors individuals’ interests and self-development. According to one 2021 DIY Biology Community Survey participant, DIY biology is thought to bring “science back to its roots of exploration” while “answering personal questions and learning more along the way.”

These visions are worth exploring, as they invoke an influential figure: the “modest witness.” The “modest witness” is a concept developed through Simon and Schaffer’s work (Shapin and Schaffer 2011), which feminist philosopher Donna Haraway formalizes and critiques (Haraway 2018). Haraway’s work is a reaction Shapin and Schaffer’s witness, who was a wealthy, privileged gentleman. Haraway argues that the scientific observer is not an objective, neutral observer of the world, but rather a situated, partial, and embodied participant in the process of knowledge production (Haraway 1988). Haraway critiques the modest of the gentleman observer and asks who gets to be modest and whose presence in science can count as modest. This idea resonates with DIY biologists’ self-awareness in shaping their own research agendas, as they recognize that scientific inquiry is always shaped by social, cultural, and personal factors.

In her work, Haraway emphasizes the importance of acknowledging the situatedness and partiality of scientific knowledge and of recognizing the ways in which scientific observation and experimentation are entangled with cultural, historical, and political factors.

First, community members’ emphasis on individual freedom and the exploration of personal interests in DIY biology indicates an awareness of the situatedness and partiality of scientific knowledge. Rather than approaching scientific enquiry a neutral and objective pursuit, DIY biologists acknowledge the ways in which their own perspectives and interests shape the questions their research questions and scientific methods.

Second, the focus on community-led learning in DIY biology reflects a recognition of the entanglement between social and cultural dimensions and scientific knowledge production. By emphasizing the role of informal community and peer-based collaboration in scientific inquiry, DIY biologists are challenging traditional models of scientific authority and expertise, by emphasizing the role of community and collaboration. Therefore, they may promote a more inclusive and socially aware approach to knowledge production.

Third, the emphasis on curiosity-driven exploration in DIY biology aligns with Haraway’s call for a more humble and self-reflective approach to scientific investigation. By acknowledging the contingencies and limitations of scientific knowledge and by remaining open to new questions and perspectives, some DIY biologists bear resemblance to Haraway’s “modest witness,” which may help to foster a more democratic and inclusive approach to scientific inquiry.

Another survey participant argues that personal freedom and independent exploration does not only benefit the individual but also produces better results:

I can learn it as I go. And maybe I’ll learn or create an entirely new approach that hasn’t been done in academia before because of their traditional methods and constraints. I can contribute to a project because I’m passionate about it.

In comparison to the academy, community members commonly imagine DIY biology as an opportunity to work at one’s individual pace, to be creative, to freely choose collaborators, and to work interdisciplinarily.

At the heart of DIY biologists’ quest for freedom is a wish to set their own research agendas. This is closely interlinked with the previously mentioned enthusiasm that DIY biology communities have for solving grand-scale problems. One DIY biologist voices that their experience in academia was “only working on a tiny repetitive portion of someone else’s project which I am not allowed to change or investigate new aspects of.” Another individual explains that while working in academia, they felt unsure of who the research they were working on would benefit:

DIY Biology is free of such constraints as being required to pursue only the flashiest publication worthy experiments and working overtime hours without overtime pay to keep doing something so mundane that might not see any pay-out until a few years and post-docs later. Who knows if this is working? Who knows why it’s not working? Who is this even benefitting? Just the author?

Moreover, as opposed to academia, DIY biology is thought to make research accessible to individuals independently of the qualifications they hold. Scholarly accounts acknowledge that DIY biologists often assert that every individual should be free to pursue their research interests and set their own agendas, regardless of educational and socio-economic background or institutional affiliations (e.g., Delfanti 2011; Delgado and Callén, 2017; Gorman 2011). They imagine that DIY biology allows for more freedom since membership in a DIY biology community laboratory is easily obtained and independent of academic degrees, prestige, or hierarchies. A survey participant explains that they would rather work in DIY biology than in academia as to not be “boxed into an extremely limited role due to the lack of a PhD.”

While the strong individualistic tendencies of DIY biology allow for greater personal freedom and self-development, it is not without pitfalls. One of the central problems that are associated with DIY biology is that the imagined freedom comes with a lack of institutional oversight. The identity construction of this extra-institutional science movement hinges on non-conformity; some even claim that the movement is “laden with anti-institution and anti-bureaucracy claims” (Delfanti 2012: 171). The DIY biology movement seeks to govern itself from the bottom-up through communal oversight and a loosely shared ethos, which raises immense controversy (Landrain et al. 2013). Some assert that the community’s effort to mimic “institutional attributes through communal oversight” does not compare to the “professional norms and regulations” that govern “academic and industry labs” (Gorman 2011: 427).

### Imaginary #3: socially relevant research agendas

In my 2021 DIY Biology Community Survey and the personal interviews I conducted, DIY biologists most often voice that they prefer DIY biology over institutional research because it allows them to solve problems with social relevance. One 2021 DIY Biology Community Survey participant complains that “academia increasingly operates as a business rather than as a knowledge generator.” On the contrary, they imagine DIY biology as “a field that can be self-defining in terms of goals, projects, and collaborations.” DIY biologists often address grand-scale problems, such as the climate crisis, the inaccessibility of scientific infrastructures in the Global South, the unprofitability of treatments for rare genetic defects, and rising prices for pharmaceuticals that make life-saving medications inaccessible.

The communities seek to respond to ‘grand’-scale, pressing problems, which is why it is perhaps unsurprising that at the start of the COVID-19 pandemic, DIY biologists were quick to set up the OPENCOVID19 initiative. The program attracted more than 4000 individuals seeking to develop “open-source and low-cost tools and methodologies that are safe and easy to use in response to the COVID-19 pandemic” (JOGL 2021). The hosting organization of this initiative is JOGL (Just One Giant Lab), a non-profit collective of open-source biotechnology enthusiasts seeking to make a change in the world. The makers of JOGL state that their goal “is to catalyze the collective creation of knowledge and solutions to resolve humanity’s most urgent challenges” (JOGL 2021).

Similarly, in personal interviews, DIY biologists often call for scientific agendas that benefit people. More specifically, they aim for research agendas that respond to pressing social and environmental problems and demands. In the minds of many DIY biologists, traditional research institutions are more often aligned with market demands than with social and environmental needs. The DIY biology community’s objective is to solve these overlooked problems. This way, they seek out zones of what has been defined as ‘undone science’—research that has “potentially broad social benefit” but is “left unfunded, incomplete, or generally ignored” by traditional research institutions (Frickel et al. 2010: 2; Hess 2016). For example, the ‘Open Insulin Foundation’ (2022) seeks to innovate a simpler and cheaper way of making insulin.

## Economies of extra-institutional science

How does DIY biology function vis-á-vis traditional research institutions and capitalist markets? The following section sheds light on the practicalities of extra-institutional science, specifically the movement’s attempt to challenge institutional oversight and funding regimes, market logics and supply chains. This research holds that under the current economic system, DIY biology’s principles can hardly be harmonized with the need to adequately fund and supply extra-institutional science communities, spaces, materials, and equipment. I then introduce and critically contextualize three initiatives that are oft-cited examples of DIY biology ‘success stories’, models of DIY biology innovation and entrepreneurship. I argue that DIY biology is tied up in capitalist economies and might even accelerate their functioning.

### Challenges

As I showed above, DIY biologists support and promote free scientific inquiry, an orientation toward participation, justice, and the common good. However, data show that DIY biologists struggle to put these principles into practice. My survey participants were asked about the challenges they face doing independent DIY biology work and activities. Accordingly, data show that DIY biologists’ struggle to fund their activities (73%) and to find enough time in their day to pursue DIY biology (64%). Other commonly reported issues include securing supplies (43%), not having a community laboratory nearby (38%) and difficulties regarding laws and regulations (29%).

Survey participants were given the option to write a short text about any other challenges they face. The problems and challenges they shared include (in descending order): Lack of training or knowledge, finding communities, mentors, and qualified collaborators, COVID-related problems (e.g., lab closures), and access to scientific literature/resources (see Table 2).

**Table 2:** DIY biologists’ challenges

What are some of the challenges you’ve faced doing independent DIY biology work and activities?	Multiple choice (*n* = 117) %
Difficulties paying for DIY biology equipment, supplies, membership, etc.	73
Not enough time	64
Supply issues (e.g., reagents)	43
No community lab nearby	38
Difficulties with local and national laws and regulations (biosecurity, biosafety)	29

Data: 2021 DIY Biology Community Survey

Some challenges overlap with those faced by institutional scientists. However, DIY biologists encounter these issues in the context of their non-institutional setting, which adds a layer of complexity to their experiences. Many DIY biologists undertake their work alongside paid labour, which often adds more strain compared to traditional scientists who typically have dedicated research positions within established institutions.

The pandemic has put a big strain on extra-institutional science spaces. One survey participant explains that COVID-19 has magnified the difficulties of running a community laboratory:

Cost of membership and lab equipment and reagents. Let’s not beat around the bush, this is the biggest barrier to entry, to starting a community lab, to joining a community lab, to finding a community lab, and to keep going to that community lab when it’s so far away and hard to get to before. Now that there’s a pandemic outside and it’s not even open, well... that’s just impossible now. If you’re looking to spin this question as a way to address how to improve access, it’s kind of systemic: it’s hard to start an independent DIY biology lab, hard to get people to attend it, pay for it, and keep it funded and afloat, and hard to keep going.

This statement echoes many others. Thus, in the following, I want to detail DIY biologists’ two central struggles: funding and obtaining supplies.

### Funding

The 2021 DIY Biology Community Survey undertaken as part of this research shows that funding is the biggest challenge that DIY biologists currently face. In a capitalist system, everything must create value and become a commodity. Even the open-source products and protocols that DIY biologists are developing and freely sharing, intending to advance the ‘social good’ by advancing science, have bottom lines and target markets (e.g., Ikemoto 2017). Table 3 shows that 90% of DIY biologists use their personal funds to, at least in part, finance their activities.

**Table 3:** DIY biologists’ sources of funding

How do you pay for your DIY biology activities?	Multiple choice (*n* = 118) %
Self-funded	90
Public funds or grants	25
Community MEMBERSHIP FEES	18
Revenue through paid workshops or training	13
Sponsorship (philanthropist/individuals/angel investors)	12
Crowdfunding/crowdsourcing	10
Sponsorship (industry or corporate)	9

Data: 2021 DIY Biology Community Survey

A few 2021 DIY Biology Community Survey participants report that they use their unemployment benefits to fund their DIY biology activities. One survey participant has made this experience and names the pandemic and depressed wages as a reason for their unemployment:

I wasn’t kidding about being broke and using my Unemployment Insurance to pay for my membership. This isn’t even the first time I’m sad to admit. I have the most time to actually go to this lab when I’m unemployed. Getting employed in this field was hard before the pandemic hit. And when Lab Technician wages pay only slightly more than McDonald’s, it really makes people question why they are even in this field to begin with.

Furthermore, results from the 2021 DIY Biology Community Survey show that half (50%, *n* = 53) of the participating DIY biologists rely solely on their personal funds to finance their activities. The other half uses a combination of private funds and other financial resources.

Only 25% can rely on at least some public funds or grants. The third and fourth biggest financial resources for DIY biologists are community membership fees (18%) and revenue through paid workshops and training (18%), which have taken a hit due to COVID-19-related lab closures. Relatively few DIY biologists are benefiting from sponsorships by philanthropists, individuals or angel investors (12%), crowdfunding (10%), and/or industry and corporate sponsors (9%). When asked about challenges and problems, one 2021 DIY Biology Community Survey participant aired their grievances about the lack of available funds: “DIYBIO is not on prioritized lists for investment or financing at the state level, so it is difficult to access funds.” Another survey participant explains how they try to make up for a lack of financial resources: “I couldn’t make enough money doing independent research and had to start a company.” This last statement indicates that starting a company does not necessarily have to be a goal for DIY biologists, it can simply become a necessity.

### Supply issues

Another key issue that sparked elaborate essay responses from survey participants is the issue of securing supplies. Generally, suppliers of the life science sector find themselves tasked with vetting customers according to national and regional laws and regulations. This vetting, or verification, process is tailored to institutions in the public or private sector, i.e., academic, industry, or government laboratories. Many DIY biologists do not pass this process and thus find themselves unable to secure essential supplies.

A 2021 DIY Biology Community Survey participant explains their struggle with securing supplies:

Some of the large suppliers […] can be very difficult for us to work with. They come up with their own rules for which businesses qualify to access supplies and equipment. (Does your business have a website? Does your mailing address match your shipping address? Do you have an institutional telephone number or email address? Who is your PI [principal investigator]?) For example, one supplier designated my lab as a residential address, despite it being located at a commercial biotech accelerator, and so they won’t let us order supplies. Their decision-making process is completely opaque and not based on any laws or regulations. […] It is absolutely infuriating.

The 2021 DIY Biology Community Survey data reveals that even DIY biologists in university-affiliated labs face difficulties obtaining supplies. Some circumvent this by having a community member who is also a university employee order supplies, bypassing suppliers' vetting. However, this temporary fix is not sustainable. Addressing this complex issue requires collaboration among the publics, regulators, suppliers, and community members to determine if and how extra-institutional scientists can access lab materials.

### DIY biology entrepreneurship & innovation

Scholarly accounts claim that DIY biology is co-produced with a Silicon Valley ethos to innovation that embraces entrepreneurship and rebellion (e.g., Keulartz and van den Belt 2016). Popular narratives invoke the mystical figure of the ‘garage entrepreneur,’ who will revolutionize biotechnology similarly to how biohackers have revolutionized the computer sciences in the 1980s and 1990s (Delfanti 2012; Kelty 2010; Wohlsen 2011). Similar to the hacker movement, DIY biology is thought to attract individuals that would like to pursue out-of-the-box research with peer support and quickly publicize, if not commercialize, the results (e.g., Delfanti 2013; De Lorenzo and Schmidt 2017). The DIY biology community laboratory is thought of as a site for speedy invention that stands in stark contrast with “the academic and the industrial Gotha [that] have become veritable obstacles for innovation.” (De Lorenzo and Schmidt 2017: 517).

Beyond the hype and doubts that surround the movement, DIY biologists are developing and marketing a range of products. In interviews and informal conversations, DIY biologists often bring up the same initiatives and companies. Thus, I found it worthwhile to briefly introduce three of these popular ‘DIY biology success stories’ here. These three initiatives give an insight into the different shapes that DIY biology innovation can take. This is to say that they approach DIY biology entrepreneurship with different goals and business models: Bento Bio is a crowdfunded start-up (Bento Bio 2021). Ginkgo Bioworks is what some term a ‘unicorn’—a start-up company valued at over $1 billion (Cumbers 2019). The Open Enzyme Collection is an initiative seeking to improve research infrastructures in the Global South (Open Bioeconomy Lab 2022).

UK-based Bento Bio creates simple, portable open-source DNA laboratory devices. The founders first met during an iGEM competition and funded the development of the Bento Bio lab through a Kickstarter crowdfunding campaign. They describe Bento Bio as a “small biotech company without investors” (Bento Bio 2021). In their mission statement, the Bento Bio founders articulate their goal of democratizing biotechnology. They argue that laboratories are too expensive and hard to access; however, “this lack of democratization of biotechnology has consequences. Genetic literacy is low and mistrust in science is high. We believe that by empowering everyone to do fundamental DNA lab work, we can make a difference” (Bento Bio 2021). Their company may be small, but their goals are not: “Together, we can unlock the potential of biology, by making essential techniques like PCR universal” (Bento Bio 2021).

Just like Bento Bio, Ginkgo Bioworks originally started as an iGEM project. The Boston-based company is developing a genetic engineering platform that utilizes synthetic biology for industrial applications, aiming to program cells to produce bio-industrial products, such as agricultural fertilizers or petroleum (Ginkgo Bioworks 2023; Nanalyze 2021). The company began with a DIY biology ethos but quickly transitioned from a university-based iGEM project to a biotech start-up, amassing over 30 patents and adhering to a traditional intellectual property regime (CBInsights 2022). Ginkgo Bioworks has received glowing press coverage and high valuations, reaching $15 billion in 2021 (Betuel 2021), although some doubt the company’s worth (Nanalyze 2021). In late 2021, Ginkgo Bioworks faced an investigation for fraudulent activity (Bloomberg 2022). While initially avoiding shareholders, the company moved from university to industry, not strictly qualifying as extra-institutional science. However, due to their proximity to the movement, Ginkgo Bioworks is still discussed under the umbrella of DIY biology (DeLorenzo and Schmidt 2017).

Based in Cambridge (UK), Ghana and Cameroon, the Open Enzyme Collection seeks to make biotechnology more accessible for researchers in the Global South (Open Bioeconomy Lab 2022). The team wants to supply the scientists and laboratories of the Global South with essential supplies, specifically enzymes. The primary funder is the Shuttleworth Foundation, a philanthropic organization that supports social change projects (Shuttleworth Foundation 2022). This project could potentially serve enormous markets of the Global South but is not strictly profit-driven.

What all three initiatives have in common is their goal to improve the world through biology, whether it is to yield the potential of synthetic biology (Ginkgo Bioworks) or to democratize biology by making it more accessible (Bento Bio, Open Enzyme Collection). For two of them, iGEM competitions were the company’s initial starting point.

All of the above-mentioned initiatives operate within capitalist bio economies: All of them have target markets, bottom lines, and depend on the same supply chains as traditional institutions. The key differences lie in their ownership, intellectual property regimes, and funding structures. The previous section showed that finances are the most common challenge for DIY biologists. The individuals that are running these three initiatives have all found different funding sources: Philanthropic grants and online crowdfunding are both two relatively new funding avenues. Both—charitable funds and crowdfunding—can be reconciled with DIY biologists’ attempt to create an alternative, open-source economy. However, as seen in the case of Ginkgo Bioworks, extra-institutional science does not always promote alternative economic principles and can just as easily cater to capitalist markets.

### Toward an alternative bioeconomy?

While some claim that DIY biology has the potential to create alternative economic principles (e.g., Carlson 2005), I want to caution against these claims and argue that DIY biology is tied up in capitalist economies and might even accelerate their functioning. ‘Unicorn’ firm Ginkgo Bio can even be categorized as a venture capitalist success story. Meyer (2012) asserts that DIY biology establishes an alternative, non-market economy, which he terms “citizen bioeconomy” (Meyer 2012: 3). The latter is constructed in opposition to the ‘closed economies’ of large corporations and the biotechnology industry, also referred to as ‘Big Bio’ (Meyer 2013: 3) and instead comprises “open, collective, distributed, and accessible” knowledge infrastructures. According to other accounts, DIY biology’s key concerns include Big Bio’s monopolies that are “based on intellectual property rights, capital-intensive laboratories and scientific expertise” (Delfanti 2013: 128). Others assert that the movement intends to challenge the industry’s concentration of power by establishing a system that favours the best ideas and is not governed by rigid intellectual property (IP) right regimes, corporate interests, and capital accumulation (De Lorenzo and Schmidt 2017; Keulartz and van den Belt 2016).

To what extent an alternative bioeconomy, with a non-proprietary knowledge control regime at its core, challenges traditional market systems is contested. Some scholars argue that the movement does not, and could not, occupy a space outside of market economies (e.g., Meyer 2013, 2014; Delfanti 2013; Ikemoto 2017). This is because capitalism leads to the concentration of resources and power. DIY biologists depend on funds that are tied up in corporate capitalism. Even an extra-institutional science movement like DIY biology depends on markets to acquire tools and products.

DIY biology is sometimes envisioned as a site where the relationship between institutional science, markets, and society is reformulated, ideally shifting the focus from capital accumulation to “knowledge creation premised on participation and justice” (Ikemoto 2017: 539). In many ways, this extra-institutional science movement can be thought of as an indicator of accelerating market economics. DIY biology fosters “an individualistic culture of openness both in information circulation and in capitalist competition, a new open frontier for science entrepreneurship” (Delfanti 2011: 56). “Collective, peer-produced biology” might not contradict but set the stage for a neoliberal open innovation paradigm (Delfanti 2011: 56). The open innovation model presents a porous environment where start-ups generate ideas that larger firms tap into. The proposed non-proprietary control regime would completely tear these boundaries down. In the community laboratories, ideas are generated in an open community setting and shared with passers-by on open lab nights. The movement’s actual impact may be diversification in scientific knowledge production by incorporating a multitude of actors and institutions, including universities, start-ups, corporations, or citizen initiatives (Delfanti 2012).

Some argue that this culture of open sharing is not rooted in selfless goals but entrepreneurial aspirations; in a strategy to corrode Big Bio’s monopolies (Delfanti 2012: 171). In fact, DIY biologists’ aspirations bear some resemblance to a techno-optimistic ‘tech for good’ approach, as some initiatives are not only aiming to create collective benefit but also produce a profit. The concept of ‘tech for good’ refers to the use of technology to target social, economic, and environmental issues, thereby promoting social good. While the concept has gained some traction, there are also serious limitations and drawbacks (e.g., Noble 2018).

There is potential for unintended consequences in DIY biology. While it may address social problems, it can also exacerbate existing ones. DIY biology, with its expert heavy membership, may reinforce power imbalances and marginalize communities. Projects driven by privileged individuals could misalign with the needs of the target community or reinforce social, political, and economic inequalities. This approach often oversimplifies social problems by ignoring the complex interplay of social, economic, ecological, and political factors.

Ikemoto (2017) argues that DIY biology is both grounded in and guided by a neoliberal ethos, making it well suited to meet industry needs. Instead of opposing market principles, DIY biology may foreshadow the market’s future transformations (Delfanti 2014). Ikemoto suggests that a “lack of robust ethos leaves DIY biology ripe for capture by biotech” (Ikemoto 2017). Rather than creating a ‘citizen biotech economy’ or overturning the system, DIY biologists might open a profitable market frontier where small, open-source products can compete with Big Bio (Delfanti 2013; Ikemoto 2017). Recent literature further explores DIY biology’s relationship to markets and its role in the evolving bioeconomy. Meyer and Wilbanks (2020) examine how DIY biology intersects with market valuations and economic principles, while Santos (2023) provides insights into the broader implications of community science labs within these market dynamics.

## Conclusion

This article provides empirically grounded insights into how DIY biologists envision their movement relative to traditional research institutions. By analyzing data from the 2021 DIY Biology Community Survey and personal interviews, I conceptualize DIY biology as extra-institutional science.

My findings highlight that DIY biologists carve out unique spaces and identities beyond traditional academia and industry but still depend on these institutions for employment, training, and resources. Survey participants expressed dissatisfaction with neoliberal academia, positioning DIY biology as a reaction to the challenges posed by neoliberalization.

The article introduces three socio-technical imaginaries derived from thematic analysis. First, DIY biology is viewed as creating spaces for socially relevant research, applying academic knowledge to societal issues. Second, it seeks to democratize science by making research infrastructures more accessible and fostering open-source knowledge. Third, it promises greater intellectual freedom and agency through curiosity-driven exploration, free from institutional constraints.

These imaginaries underscore DIY biology’s roots and missions, revealing inherent tensions as it engages in boundary work to define its own identity. The potential for DIY biology to become institutionalized, forming a fourth site of scientific inquiry alongside academia, industry, and government labs, is an area for future exploration.

Additionally, the article sheds light on the practical challenges faced by extra-institutional science, including issues with oversight, funding, and supply chains. Survey data reveal that DIY biologists often struggle with financing and obtaining lab supplies, suggesting a need for regulatory attention.

The concept of extra-institutional science opens avenues for future research and policy development. It provides a framework for understanding how scientific practices outside traditional institutions can evolve and interact with established structures and institutions. This concept can be used to explore how these communities negotiate their roles and responsibilities, develop new forms of collaboration, and influence broader scientific and societal practices. The analysis of three exemplary initiatives shows varying models of innovation and highlights the critical role of alternative funding sources like charitable grants or crowdfunding. Currently, DIY biology remains entangled with capitalist economies, potentially influencing its development and sustainability.

## Data Availability

I would like to confirm that the manuscript comprised original material that is not under review elsewhere. The study on which this research is based has been subject to appropriate ethical review and has been approved by the Ethics Committee at the University of Cambridge, Department of Sociology. All research participants have given written consent prior to their participation. Additionally, there are no competing interests, intellectual or financial, in the research detailed in the manuscript. This research was funded by the ESRC (Economic Social Research Council) and the University of Cambridge.

## Footnotes

1 However, demographic analysis of citizen scientists show that many participants are more often highly educated, more affluent, more middle aged, and predominantly white (Committee on Designing Citizen Science to Support Science Learning et al. 2018).
